# Gradual Colonic Impaction of a Chicken Bone Associated with Inflammatory Pseudotumor Formation and Nonocclusive Colon Ischemia

**DOI:** 10.1155/2014/215465

**Published:** 2014-02-11

**Authors:** Stefania Fosi, Simone Altobelli, Alessio Bindi, Massimo Villa, Flavio De Sanctis, Mauro Montuori, Edoardo Ricciardi, Piero Rossi, Giuseppe Petrella, Giovanni Simonetti

**Affiliations:** ^1^Department of Diagnostic Imaging, Molecular Imaging, Interventional Radiology and Radiotherapy, Policlinico Tor Vergata (PTV), Viale Oxford 81, 00133 Rome, Italy; ^2^Department of Experimental Science and Surgery, Policlinico Tor Vergata (PTV), Viale Oxford 81, 00133 Rome, Italy

## Abstract

Foreign body (FB) ingestion is a common clinical problem and most FBs pass through the gastrointestinal tract without the need for intervention. A wide spectrum of clinical presentations may be possible and these can be either acute or chronic. We present a case of an 83-year-old woman featuring insidious abdominal discomfort who was hospitalized in our institution due to worsening symptoms. She underwent contrast-enhanced computed tomography (CT) evaluation which showed the presence of a significant parietal thickening of the transverse and descending colon, a mesenteric loose tissue imbibition, venous engorgement, and no filling defect of visceral arteries, suggesting a condition of nonocclusive colon ischemia. A hyperdense FB was identified in the sigma and was associated with a small pseudotumoral mass. The patient underwent surgical exploration which confirmed the hypoperfusional state of the colon, showing the presence of a chicken bone perforating the sigma and lying in the context of a pseudotumoral mass. Our experience shows how contrast-enhanced CT is feasible and can be strongly recommended as a first-line imaging tool on suspicion of colon ischemia and also how it can easily identify the underlying cause, in our case a FB sealed perforation of the sigma with pseudotumoral mass formation.

## 1. Introduction

Foreign body ingestion and food bolus impaction commonly occur, and while more than 80% of foreign objects pass through the gastrointestinal (GI) tract without the need for intervention, 20% and 1% of them will, respectively, need endoscopic or surgical procedures. The most frequently ingested foreign bodies in adults are fish bones (9–45%), bones (8–40%), and dentures (4–18%). Ingested foreign bodies mostly cause nonspecific symptoms; however, when accompanied by GI perforation, patients may complain of abdominal pain (95%), fever (81%), or localized peritonitis (39%). Imaging plays an important role in the diagnostic process of both FBs localization and identification of related complication such as perforation and bowel ischemia. While other imaging techniques lack sensitivity and specificity, contrast-enhanced CT with postprocessing reconstructions is of choice permitting achieving a good grade of accuracy.

## 2. Case Presentation

**Figure 1:**
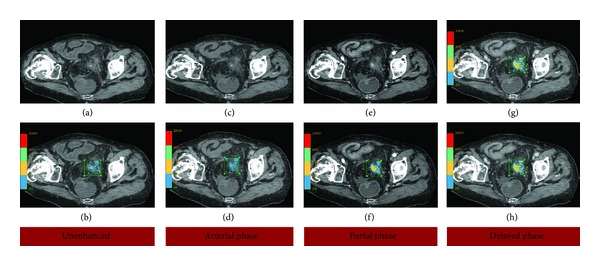
Oblique CT images before ((a), (b)) and after contrast medium administration in the arterial phase ((c), (d)), portal phase ((e), (f)), and delayed phase ((g), (h)) showing the foreign body and perifocal contrast-enhancement. In the lower images ((b), (d), (f), and (h)) a density color map has been superimposed using Advantage Workstation 4.5 (GE) to better depict the increase of HU in the different phases.

**Figure 2:**
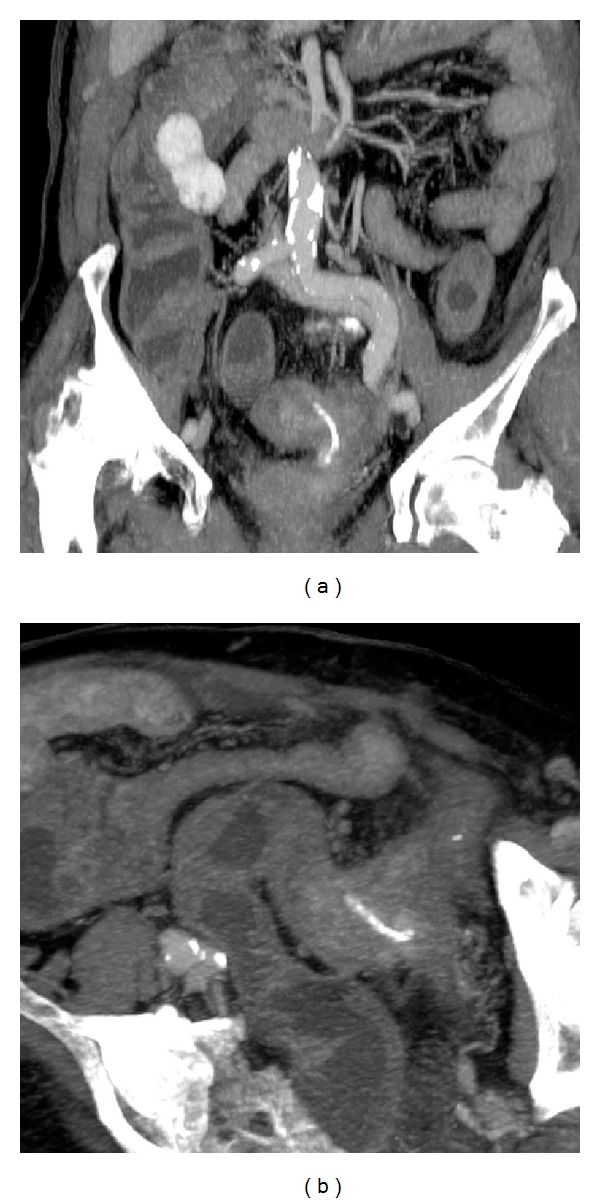
CT MPR images after contrast medium administration in portal (a) and delayed phase (b) showing the curvilinear sharp-pointed foreign body in the sigma and the contrast-enhancement within the inflammatory pseudotumoral mass at the FB tip.

**Figure 3:**
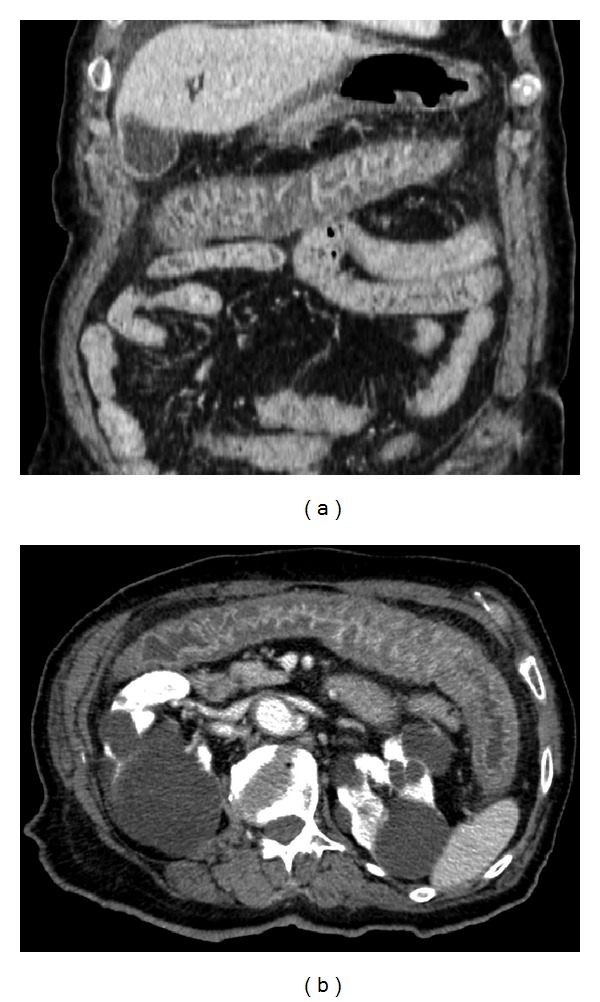
MPR CT images documenting parietal thickening and venous engorgement of the transverse colon (a, b).

**Figure 4:**
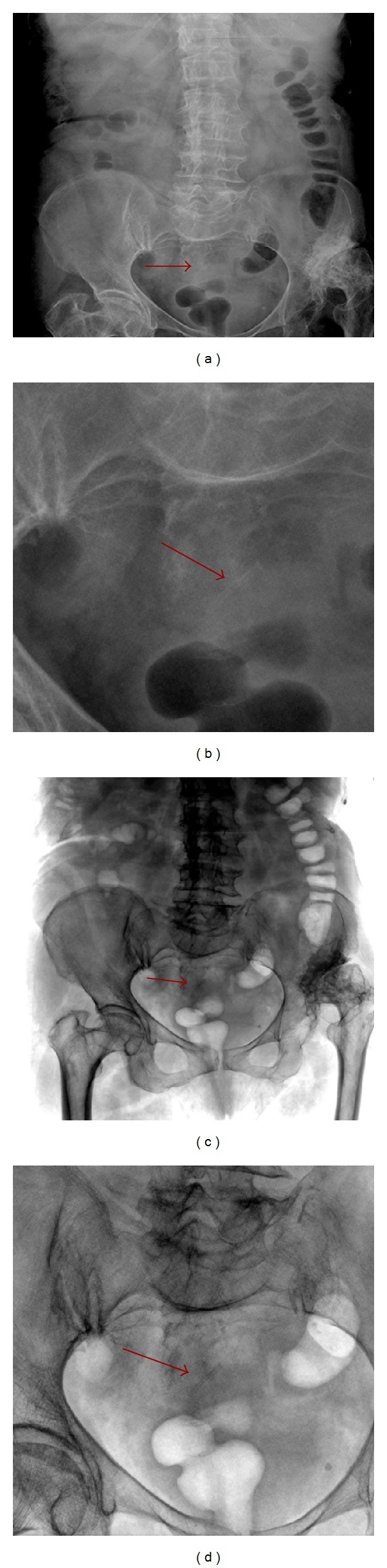
Plain digital radiographs made in January previous hospitalization in another institution and evaluated again in May after window stressing (a, b) and with inversion effect (c, d) showing that the foreign body was already present but not easy to be demonstrated without clinical suspicion due to its low radiopacity.

**Figure 5:**
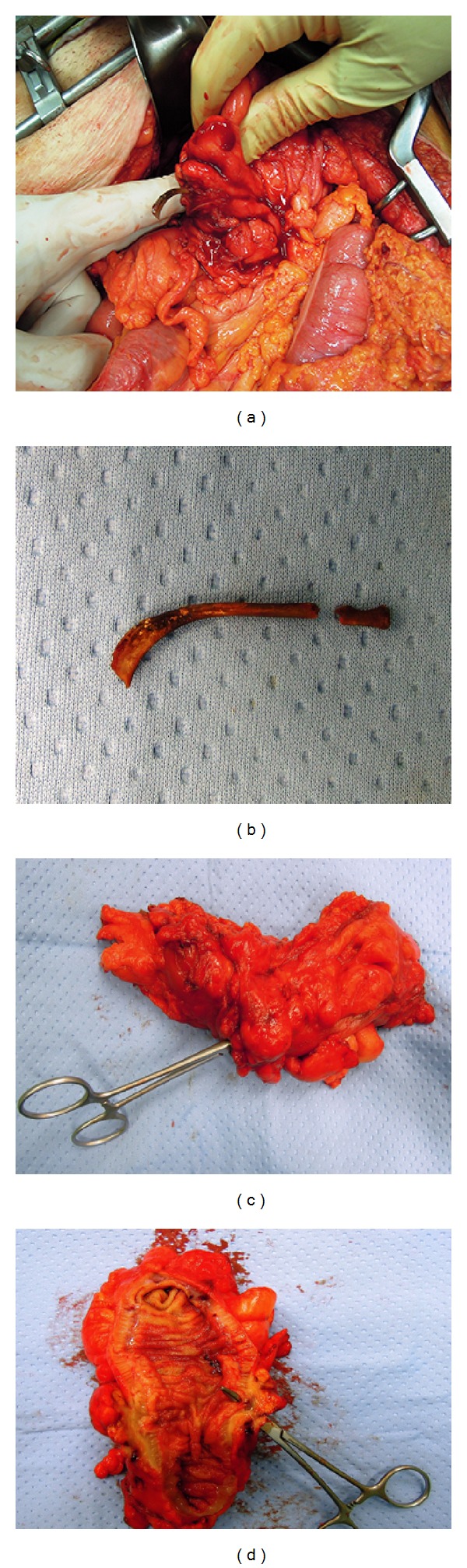
Intraoperative pictures showing the foreign body perforating the pseudotumour, (a) chicken bone, (b) resected colon with evidence of the fistula, and the thickening of the wall (c, d).

**Figure 6:**
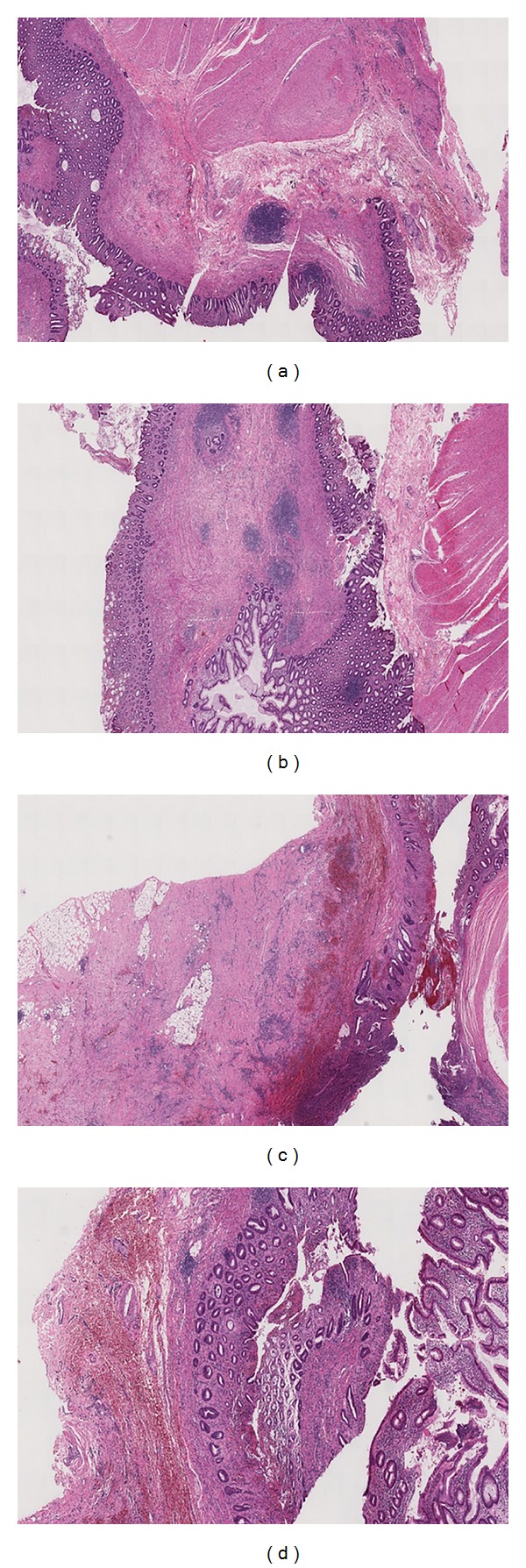
Microscopic view showing the ischaemic aspects and the presence of an hemorrhagic ulcer in the colonic wall.

An 83-year-old woman was admitted to the emergency department of our hospital on May 5 with a clinical history of abdominal pain, nausea, vomiting, and diarrhea which started three days previously. Her past medical history was characterized by chronic atrial fibrillation, cardiomegaly, and a not-further-specified adnexal mass asportation. The patient denied being a smoker and referred to chronic assumption of digitalis, furosemide, and oral anticoagulant therapy. She also referred to a previous hospitalization in January in another hospital with similar but milder symptoms, resolved in few hours. At that time a plain abdominal X-ray was performed and the radiologist's report was negative. We could not examine the X-ray film prior to performance of CT as it was brought later. At physical examination, she had no fever, with a painful and tender abdomen at superficial and deep palpation on the mid-lower quadrants. Blumberg's, Murphy's, and McBurney's signs were negative. Peristalsis was torpid. Digital rectal exploration showed fecal traces and blood streaks. Blood gas analysis showed a pH of 7.38, pCO_2_ of 29 mmHg, pO_2_ of 84 mmHg, Na^+^ of 139 mmol/L, K^+^ of 3.5 mmol/L, Cl^−^ of 104 mmol/L, and Lactates of 3.0 mmol/L and SpO_2_ of 94.7%. Blood test results were as follows: Hb 17.60 g/dL, hematocrit 51.90%, white blood cell count 22.710 *μ*L (neutrophilia 83.3%), PT-INR 1.78, glucose 158 mg/dL, blood urea 54.00 mg/dL, and creatinine 1.50 mg/dL. Upon clinical suspicion of bowel obstruction or infarction, the patient underwent contrast-enhanced CT examination (LightSpeed VCT 64 Slice GE; slice thickness: 1.25; contrast media: Iomeron 350 mg/mL Bracco) which showed the presence of a significant parietal thickening of the transverse and descending colon, a mesenteric loose tissue imbibition, venous engorgement, no filling defect of visceral arteries, and the presence of fluid collections in the Douglas pouch and perihepatic space. As a collateral finding, a hyperdense sharp-pointed curvilinear foreign body was identified in the sigma and was associated with a perifocal persistent contrast-enhancement (CE) within a pseudotumoral mass (Figures 1, 2, and 3). It was not possible to differentiate whether it was lying in the bowel lumen or in the context of the intestinal wall. No free abdominal air was discovered. Having previously evaluated the CT scan and examining the X-ray that the patient's family had brought in the meantime, our radiologist concluded that the FB was already present in January (Figure 4). Due to worsening of the clinical condition and on the basis of imaging findings, it was decided to perform an emergency surgical intervention. After laparotomy was performed, 100 mL of citrine fluid was collected and sent to our laboratory to be analyzed. Macroscopically small bowel loops and in particular the colon were pale, suggesting a condition of hypoperfusion, although no marked areas of ischemia and infarction were found. Pulsation of the superior mesenteric artery was present. The distal portion of the sigmoid colon resulted tenaciously adherent to the pelvic wall, with a wooden consistency. An intraoperative endoscopy was performed, but it was not possible to pass beyond the rectal-sigmoid junction due to a stenotic condition related to the rigidity and angle of the sigma wall. Dissection of the sigma from the pelvic wall revealed an ingested chicken bone perforating the sigma and lying within an inflammatory pseudotumour originating from the colonic wall (Figure 5). A Hartmann's intervention was performed, consisting of the resection of the rectosigmoid colon with closure of the rectal stump and an end colostomy. This surgical procedure was decided in order to avoid the risk of dehiscence of a colocolic anastomosis due to the evident intestinal hypoperfusion. A control CT, performed 8 days after surgery, did not show any complication or fluid collection. Histological examination showed extensive hemorrhagic ulceration of the colonic mucosa, complicated by the perforation of the colonic wall and associated serositis. Surrounding areas presented ischemic aspects with reactive locoregional lymphnodes. Collected peritoneal fluid analysis revealed the presence of reactive mesothelial cells, blood cells, and amorphous proteinaceous material (Figure 6). The patient was dismissed 12 days after the operation in good status. Regular checks have been carried out in the outpatient setting.

## 3. Discussion

Foreign body ingestion and food bolus impaction commonly occur and while more than 80% of foreign objects pass through the gastrointestinal (GI) tract without the need for intervention, 20% and 1% of them will, respectively, need endoscopic or surgical intervention [1, 2]. It is more common in the pediatric and elder populations or in adults affected by psychiatric disorders, alcohol intoxication, and developmental delay. Previous studies have shown that preexisting pathology of the GI tract such as strictures, malignancies, oesophageal rings, achalasia, Meckel's diverticulum, and diverticulosis can promote impaction [3–5]. The most frequently ingested foreign bodies in adults are fish bones (9–45%), bones (8–40%), and dentures (4–18%). It is mandatory, where possible, to categorize foreign objects by dimension, morphology, and surface and material or chemical composition, which may reflect the intrinsic radiodensity and the possibility of complication, which is higher (35%) for sharp-pointed objects as in our case [6]. GI tract perforations due to a foreign body have been reported in all segments but tend to occur at sites of acute angulation, such as the ileocecum or rectosigmoid junction [7]. Perforation can occur acutely or gradually and foreign bodies may take several paths, including lying in the bowel lumen or wall and migrating to distal organs or the abdominal cavity. It follows that a wide spectrum of clinical presentation may be possible and be either acute or chronic. Gradual perforation with chronic symptoms usually occurs in the stomach or in the large bowel, in relation to the thicker wall and the close proximity of the omentum and adjacent organs, which may seal the perforation site [8, 9]. Ingested foreign bodies mostly cause nonspecific symptoms [10]. However, when accompanied by GI perforation, patients may complain of abdominal pain (95%), fever (81%), or localized peritonitis (39%). Clinical presentation also includes generalized peritonitis, abscess formation, perineum and scrotal abscess, enterovescical fistulas, obstruction, and haemorrhage. Patients often do not remember having ingested the implicated foreign body found during radiological investigation or surgery [11]. Colon ischemia (CI) is caused by a severe reduction of blood supply such that the metabolic demands are not met. It includes a wide spectrum of injury, including reversible colopathy, transient colitis, chronic colitis, gangrene, strictures, and fulminating universal colitis and it often occurs in the transient subtype in elderly people with an average age of 70 and with several predisposing conditions [12, 13]. As mesenteric ischemia, it may present an occlusive or nonocclusive nature, although the true former type is rarely observed. In our case, the patient presented with at least five known predisposing conditions (cardiac failure, IBD, FA, digoxin, and diuretics assumption) [14]. CI is usually segmental, involving the left colon in 80% of cases (splenic flexure at Griffith's point: 25% and sigmoid junction at Sudeck's point: 55%) and the right colon in 10–25% of cases and entire colon in less than 10% of cases. Mandatory for therapeutic planning is the distinction between severe CI with transmural involvement and gangrene, with or without associated multiple organ failure (MOF), and mild-moderate CI with limited mural involvement. CI symptoms are nonspecific and usually consist of abdominal pain, lower GI bleeding, tenderness, and sometimes nausea and vomiting due to the eventual associated ileus. CI can mimic diverticulitis and intestinal pseudoobstruction. Although being lacking in sensitivity and specificity, laboratory markers can be useful to investigate suspected CI. Imaging plays an important role in the diagnostic process of CI and usually permits the identification of foreign body and related complications in the GI tract. Regarding foreign body detection, several imaging modalities, such as plain radiography, ultrasonography, CT, and metal detectors have been used over time. Radiography of the abdomen, or of the suspected region of impaction, may be used as an initial screening method, although it fails in the identification of radiolucent objects and is unreliable in detecting fish bones and small chicken bones due to their low density, which may be concealed by fluid or tissue mass. As stated above, our patient was evaluated for similar but milder symptoms more than three months previously, with plain abdominal X-ray, but the presence of the foreign body, supposedly already there, was undetected. Another cause of the foreign body nondetection at that time was the incomplete knowledge of the radiologist, as no history of accidental ingestion was referred. Previous studies show overall sensitivity of 32% and specificity of 91% in detecting fish bones [15]. Some advantages in detecting low density objects can be achieved using 70 Kv instead of 90 Kv. Administration of a contrast medium should be avoided due to the risk of aspiration, the possible presence of a perforation, the reduced ability to assess the mucosa, and the possible masking of the foreign body [16]. Pneumoperitoneum is rare, as the foreign body is gradually impacted and the perforation site is covered by inflammatory tissue. Ultrasonography is not commonly used to identify foreign bodies in the GI, as documented by the low number of publications in the literature. Sonography, unlike conventional radiography, is not dependent on radiographic density and does not involve ionizing radiation, but it has been considered a poor imaging modality for detection of abdominal foreign bodies and in particular objects localized in the bowel due to air artefacts. With or without administration of intravenous contrast medium, CT is the technique of choice when low density foreign bodies are suspected. Previous studies have reported sensitivity of 100% with specificity of 91%. Potential limitations of CT examination are the lack of observer knowledge, the slice thickness, and the presence of motion artifacts. According to the literature the best results in terms of sensitivity are achieved using a multidetector computed tomography (MDCT) with 1.25 or 0.65 slice thickness and examination of the images obtained with multiplanar reconstructions (MPR). Oral contrast medium should be avoided for the same reasons mentioned above regarding radiography, whereas endovenous contrast is recommended when any complication due to foreign body impaction is suspected, although sometimes fish bone can be mistaken for blood vessels. At last, metal detectors can successfully be applied in the search for metal foreign bodies, especially in children, and some studies have shown sensitivity of 94% and specificity of 100% [17]. Regarding CI, several imaging modalities are available such as plain radiography, barium enema, CT, and in some cases mesenteric angiography. Plain radiography can demonstrate only nonspecific signs such as mural thickening, bowel distension, aperistalsis, and thumb printing in only 21% of patients [18]. In the case of perforation, free abdominal air or air in the portal vein might be seen. Barium enema may demonstrate thumb printing in 75% of cases. Ulcers, ridges, strictures, and sacculations can also be seen but are nonspecific signs [19]. Barium enema should be avoided in case of perforation and makes the later use of endoscopy or angiography more difficult. Contrast-enhanced CT is being used more and more routinely nowadays when assessing patients with nonspecific abdominal pain. CT imaging technique on suspicion of CI theoretically requires administration of both oral and rectal contrast material or water and intravenous contrast injection during the examination. We did not perform rectal administration of contrast agent due to suspicion of gut perforation. CT can identify complications and exclude other medical conditions, narrowing the differential diagnosis options. It can easily demonstrate wall thickening in 80% of patients, with “double halo” or “target” signs, thumb printing, pericolonic fat stranding, peritoneal fluid, and the site of obstruction of the mesenteric vessels in case of occlusive CI. These findings can be identified also in the case of nontransmural CI, as in our case. In the case of bowel infarction, air in the portal vein, or in the bowel wall, may be seen. According to some studies in the literature, mural pneumatosis has been identified in 71% of cases with transmural CI [20–23]. Sonography may help in the differential diagnosis between CI and inflammatory bowel disease but is often affected by artifacts and is operator-dependent. We attempted to perform ultrasonography, failing to visualize the colon wall, due to air artifacts and the habitus of the patient. Mesenteric angiography has no place in the diagnosis or treatment of CI, as opposed to acute mesenteric ischemia. The presence or absence of angiographic abnormalities does not seem to correlate with the disease prognosis. Angiography may be needed in some cases to allow the distinction between acute mesenteric ischemia and CI to be apparent, or when only the ascending colon is involved, so as to rule out an obstruction of the superior mesenteric artery [24]. Other techniques, such as scintigraphy, are not used in our emergency department. To conclude, our experience shows how contrast-enhanced CT is feasible and can be strongly recommended as a first-line imaging tool on suspicion of colon ischemia and also how it can easily identify the underlying cause, in our case a FB sealed perforation of the sigma with pseudotumoural mass formation.
